# Stroke recurrence is associated with unfavorable intracranial venous outflow in patients with symptomatic intracranial atherosclerotic large vessel severe stenosis or occlusion

**DOI:** 10.3389/fneur.2023.1156315

**Published:** 2023-05-09

**Authors:** Jiali Gao, Liang Zhang, Jiaxin Lin, Jiajie Yang, Mingzheng Yao, Zhongyuan Cheng, Xiangran Cai, Li’an Huang

**Affiliations:** ^1^Department of Neurology, Clinical Neuroscience Institute, First Affiliated Hospital of Jinan University, Guangzhou, China; ^2^Medical Imaging Center, First Affiliated Hospital, Jinan University, Guangzhou, China

**Keywords:** cortical vein, collaterals, intracranial atherosclerotic disease, stenosis, perfusion

## Abstract

**Objective:**

The purpose of this study was to investigate the predictive value of intracranial venous outflow for recurrent cerebral ischemic events (RCIE) in patients with symptomatic intracranial atherosclerotic large-vessel severe stenosis or occlusion (sICAS-S/O).

**Methods:**

This retrospective study included sICAS-S/O patients with anterior circulation who underwent dynamic computed tomography angiography (dCTA) and computed tomography perfusion (CTP). Arterial collaterals were evaluated using the pial arterial filling score for dCTA data, tissue-level collaterals (TLC) were assessed using the high-perfusion intensity ratio (HIR, Tmax >10 s/Tmax >6 s), and cortical veins were evaluated using the multi-phase venous score (MVS) for the vein of Labbé (VOL), sphenoparietal sinus (SPS), and superficial cerebral middle vein (SCMV). The relationships between multi-phase venous outflow (mVO), TLC, and 1-year RCIE were analyzed.

**Results:**

Ninety-nine patients were included, 37 of whom had unfavorable mVO (mVO−) and 62 of whom had favorable mVO (mVO+). Compared with the mVO+ patients, mVO- patients had a higher admission National Institutes of Health Stroke Scale (NIHSS) score (median, 4 [interquartile range (IQR), 0–9] vs. 1 [IQR, 0–4]; *p* = 0.048), larger ischemic volume (median, 74.3 [IQR, 10.1–177.9] vs. 20.9 [IQR, 5–86.4] mL; *p* = 0.042), and worse tissue perfusion (median, 0.04 [IQR, 0–0.17] vs. 0 [IQR, 0–0.03]; *p* = 0.007). Multivariate regression analysis showed that mVO− was an independent predictor of 1-year RCIE.

**Conclusion:**

For patients with sICAS-S/O of the anterior circulation, unfavorable intracranial venous outflow is a potential imaging indicator for predicting higher 1-year RCIE risk.

## 1. Introduction

Patients with symptomatic intracranial atherosclerotic stenosis (sICAS), especially those with large-vessel stenosis or occlusion, have a considerable risk of stroke recurrence (1, 2). Although dual antiplatelet medication and comprehensive risk factor management have been utilized to prevent secondary stroke, the incidence of recurrent stroke in individuals who present with stroke associated with symptomatic intracranial atherosclerotic large-vessel severe stenosis or occlusion (sICAS-S/O) exceeds 20% at 1 year (3–5). Better indicators are urgently needed to help identify high-risk subgroups and guide clinical decision-making.

In addition to plaque stability, cerebral perfusion status is also considered an important factor that affects stroke recurrence in patients with sICAS-S/O. Previous studies have shown that inadequate perfusion is a predictor of ischemic stroke recurrence (6–8), and cerebral perfusion assessment may assist in recognizing patients at increased risk of recurrent stroke (9). Prior studies have primarily used arterial collateral status to infer tissue-level perfusion in patients with sICAS, but cerebral perfusion is influenced by the arteries that supply brain tissue as well as the veins draining from the brain (10–12). Arterial collateral assessment alone is not equivalent to tissue-level collaterals (TLC), and arterial collateral status does not accurately reflect tissue perfusion. At present, cerebral perfusion imaging, such as computed tomography perfusion imaging (CTP) and magnetic resonance perfusion-weighted imaging, are widely used in clinical practice, but these methods are expensive and expose patients to radiation and contrast chemicals (13, 14). Accurate assessment of perfusion status, in addition to being influenced by the upstream artery and perfusion itself, is also related to venous outflow. Previous studies have found that excellent downstream venous outflow reflects good tissue perfusion and collateral arteries status, which serve as upstream arteries in acute ischemic stroke patients with large-vessel occlusion (15). Intracranial venous outflow is useful for predicting the prognosis of acute ischemic infarction (16–19). However, its role in predicting recurrent cerebral ischemic events (RCIE) remains unclear. Of particular relevance is its potential as a screening indicator for sICAS-S/O patients with a high risk of stroke recurrence.

In previous studies, the prognostic evaluation on cortical vein score difference in stroke and cortical vein opacification score (10, 20) were used to semi-quantify the state of venous drainage. These methods assess drainage status by scoring venous opacification based on single-phase computed tomography angiography (CTA) in patients with acute ischemic stroke. However, opacification of cortical veins on single-phase CTA does not accurately reflect the velocity of venous outflow and does not account for detectable but delayed venous opacification (17). Dynamic CTA (dCTA) can display blood vessels in real time and overcomes the shortcomings of single-phase CTA.

The high recurrence of stroke in sICAS is a problem that has been urgently addressed, and the venous assessment method has been validated by prior studies, and the multiphase venous score has the advantage of comprehensive assessment compared with arterial collateralization alone (17, 21, 22). Therefore, based on previous studies on venous, we conducted this retrospective study to determine whether intracranial venous outflow is associated with TLC and whether it is valuable in predicting RCIE in patients with sICAS-S/O.

## 2. Materials and methods

### 2.1. Participants

**Figure 1:**
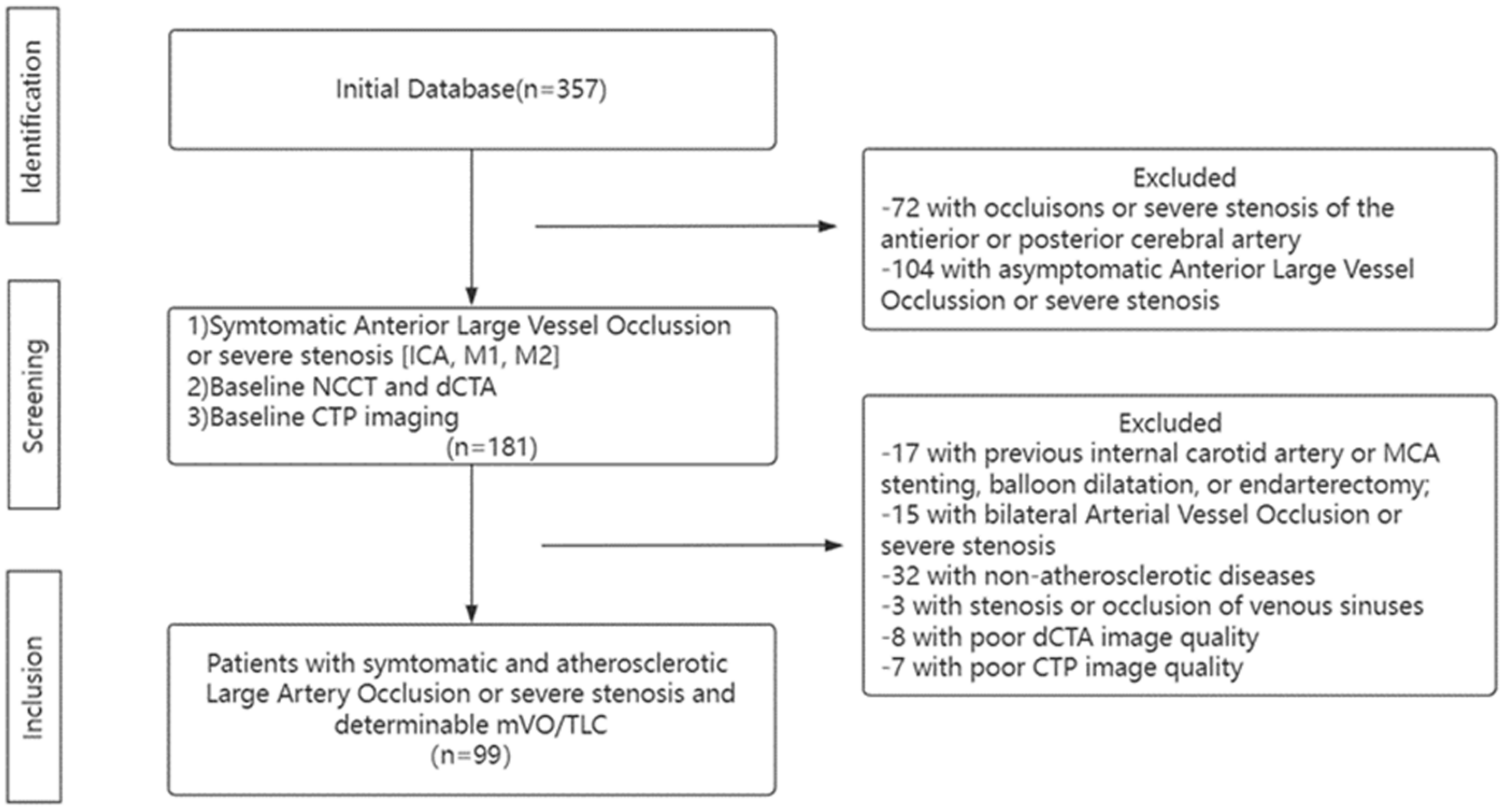
Flow Chart. ICA, Internal Carotid Artery; dCTA, dynamic Computed Tomography Angiography; NCCT, noncontract Cranial Computed Tomography; CTP, Computed Tomography Perfusion; mVO, Multi-phase Venous Flow; TLC, Tissue-Level Collaterals and MCA, Middle Cerebral Artery.

The clinical and radiological data for patients treated at the Department of Neurology of the First Affiliated Hospital of Jinan University between January 2018 and January 2022 were reviewed in this sampling study. The inclusion criteria were as follows: (1) had ischemic stroke or TIA events within 2 weeks; (2) had Trial of Org10172 in Acute Stroke Treatment classification of stroke of large-artery atherosclerosis; (3) the culprit vessel of stroke was intracranial segment of the internal carotid artery (ICA) or proximal M1 middle cerebral artery (MCA) with severe stenosis (70% ~ 99%) or occlusion which was defined according to North American Symptomatic Carotid Endarterectomy Trial (NASCET) (23); and (4) had cerebral dCTA and CTP performed within 1 week after admission. Exclusion criteria were as follows: (1) had previous ICA or MCA angioplasty or ICA endarterectomy; (2) had a contralateral artery with a serious stenosis or occlusion; (3) had stenosis or occlusion of venous sinuses; (4) had image quality issues hindering further image analysis; and (5) were unavailable for follow-up. A flow chart describing the study population selection is shown in Figure 1. The First Affiliated Hospital Clinical Ethics Committee of Jinan University reviewed and authorized the investigations involving human subjects. The participants provided written informed consent to participate in this investigation.

### 2.2. Image analysis

**Figure 2:**
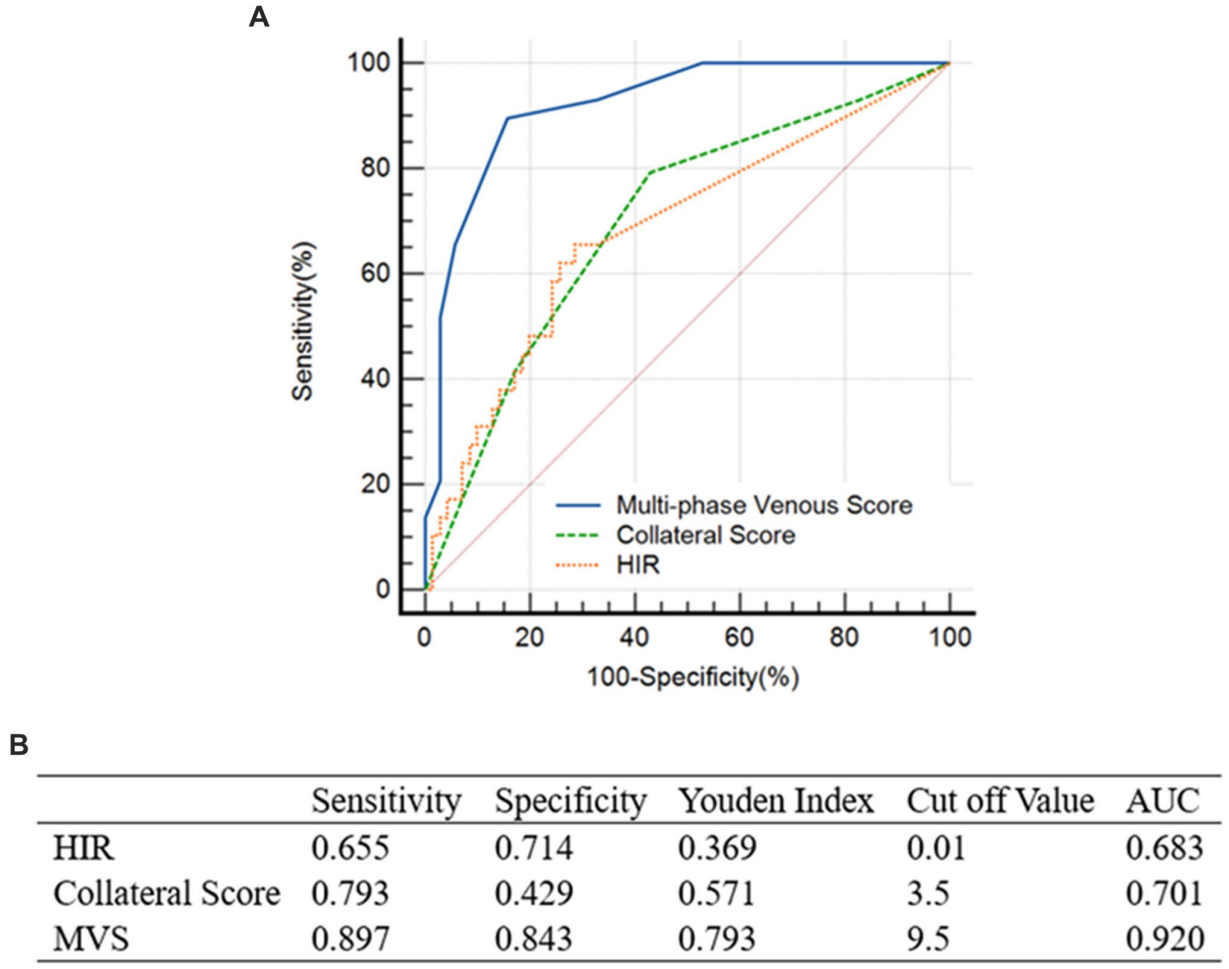
Comparison of ROC Curve. **(A)** The comparison of ROC Curve demonstrates the result when assessing MVS, collateral score and HIR against the recurrent ischemic events. Among these, area under the curve (AUC) of MVS is 0.920. Optimal cut-off for MVS was ≥9.5, (i.e., ≥10), which scored the highest on Youden’s index with a sensitivity of 89.7% and a specificity of 84.3%. **(B)** Table showed the data of ROC curve analysis.

The degree of vascular stenosis was measured on CTA by an experienced radiologist. CT perfusion studies were analyzed using F-STROKE Software (Version 1.0.9; Neuroblem Ltd. Company, Shanghai, China). The ischemic core volume was calculated automatically by F-STROKE Software, which defined ischemic core as the area of tissue where cerebral blood flow has decreased by at least 70% compared with that in the contralateral cerebral hemisphere (24). Using the HIR, TLC was calculated as ischemic brain tissue volume divided by brain tissue volume with a *T*_max_ delay >6 s. An analysis of receiver operating characteristic (ROC) data defined favorable TLC as an HIR <0.01 and unfavorable TLC as an HIR >0.01. Based on a 3-point scale, opacification of the ipsilateral vein (the occluded side of the intracranial artery) was compared to that of the contralateral vein. Ratings were given as follows: 0 – absence of ipsilateral opacification; 1 – reduced asymmetric opacification; and 2 – presence of similar or increased opacification (17). Scores were derived from all three veins: vein of Labbé (VOL), sphenoparietal sinus (SPS), and superficial cerebral middle vein (SCMV). These veins were chosen since they account for most of the venous drainage in the MCA region and have less anatomical variability compared to other cortical veins (25). Across all three phases of dCTA, the maximum venous score was 6 and the maximum multiple-phase score (MVS) was 18 (MVS and TLC are shown in Figure 2). An MVS of 10–18 was considered indicative of a favorable multi-phase venous outflow (mVO) and an MVS of 0–9 was considered indicative of an unfavorable mVO, based on ROC analysis (20). To establish inter-reader agreement, mVO was initially determined by two neuroradiologists in a subset of studies. An assessment of the collaterals on dCTA was conducted using the pial arterial filling score (26). Based on an ROC, favorable collaterals were defined as those scoring 4–5, while unfavorable collaterals were scored 0–3 (20).

### 2.3. Outcome measures

The primary outcome was the 1-year RCIE (including ischemic stroke and TIA) occurred in the stenosis or occlusion artery supply area, which was confirmed according to the clinical symptoms and diffusion weighted imaging examination. The secondary outcome was an unfavorable TLC profile, which was defined as an HIR >0.01 before treatment. We followed all patients for a period of 12 months or until death and managed their modifiable risk factors and antiplatelet therapies in accordance with secondary prevention guidelines.

### 2.4. Statistical analysis

**Figure 3:**
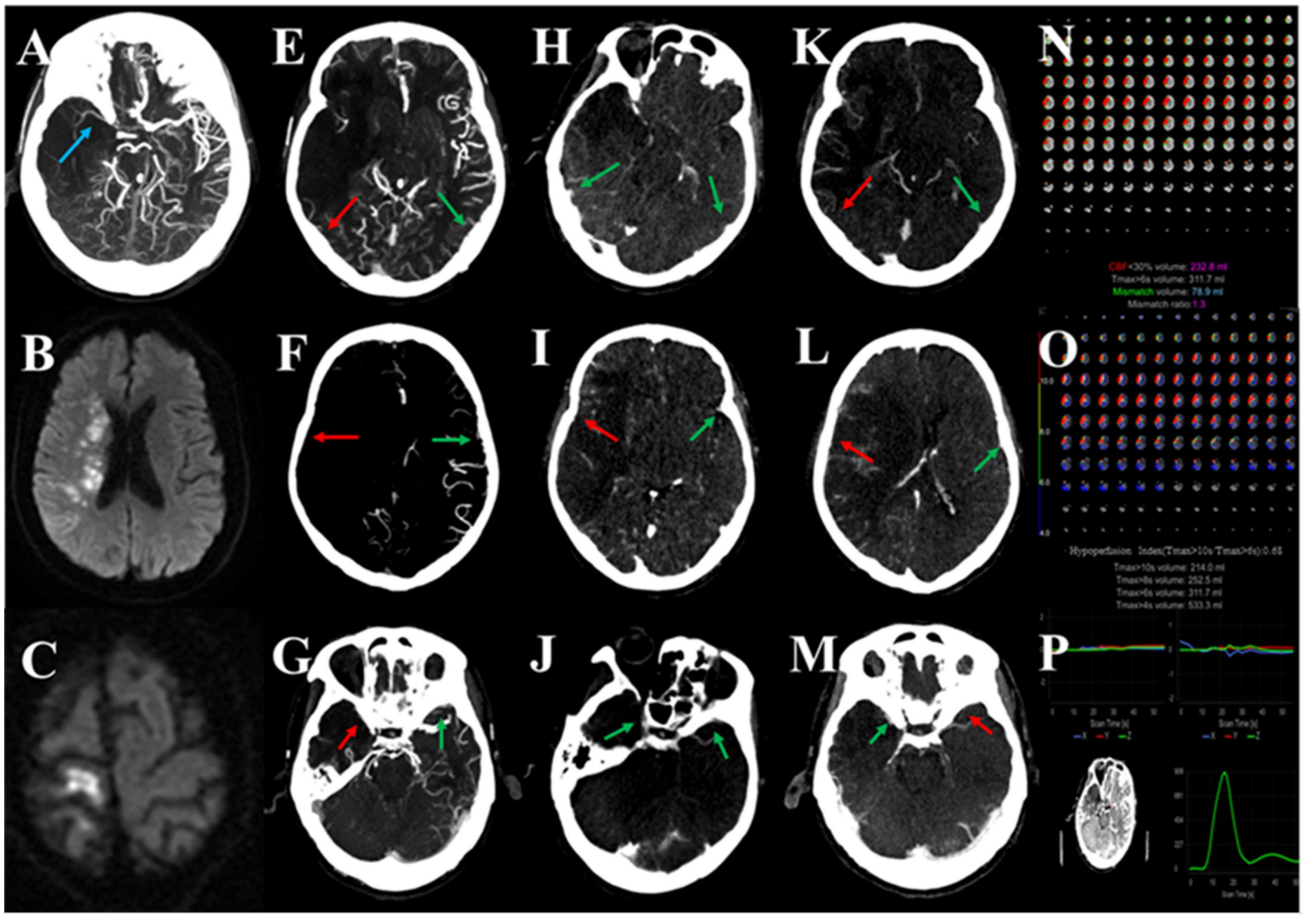
Association between multi-phase venous outflow (mVO), recurrent cerebral ischemic events (RCIE) and tissue-level collateral (TLC) in symptomatic intracranial atherosclerotic large vessel severe stenosis or occlusion (sICAS-S/O). A patient with sICAD due to occlusion of the M1 segment of the middle cerebral artery (MCA; **A**), with recurrent ischemic stroke **(C)** after the original recurrent stroke **(B)** for 6 months, with an unfavorable mVO profile **(E–M)** and unfavorable TLC **(N,O)**, and a good quality image **(P)**. Red arrows indicate poor venous contrast opacification and green arrows indicate cortical veins with moderate or good contrast filling. In this patient, unfavorable mVO (multi-phase venous outflow, multi-phase venous score = 6; **E–M**) was found to be associated with the recurrence of ischemic events. Poor venous outflow showed contrast opacification of the vein of Labbé (VOL; **E,H,K**), superficial middle cerebral vein (SMCV; **F,I,L**), and sphenoparietal sinus (SPS; **G,J,M**) in all three phases of mCTA on the corresponding infarcted right hemisphere.

In this study, statistical analysis was conducted using IBM SPSS Statistics 27.0 (IBM Corporation, Armonk, NY, United States). Data from neuroimaging, clinical factors, and patient demographics were compared between the two groups utilizing the *t*-test, *Z*-test, and Wilcoxon rank-sum test. To determine the optimal cut-off value for dichotomizing mVO, TLC, and dCTA collaterals into favorable and unfavorable groups, ROC analysis was performed with RCIE as the outcome variable (Figure 3 in the Data Supplement) (20). An analysis of the clinical and imaging variables for predicting RCIE and unfavorable TLC was conducted using multivariate binary logistic regression models. For the primary outcomes, several predefined factors were considered when calculating the regression models: age, National Institutes of Health Stroke Scale (NIHSS) score at admission, unfavorable mVO, unfavorable TLC, and unfavorable dCTA collaterals. For the secondary outcomes, the regression models were adjusted for the following prespecified factors: age, NIHSS score at admission, hypertension, and unfavorable mVO. A significance level of 0.05 was used for alpha, and all results reported are two-sided.

## 3. Results

Total 99 patients were included in our study, of which 7 were TIA, and 92 were ischemic stroke. During one-year follow up, 28.6% (2/7) TIA patients and 29.3% (27/92) ischemic stroke patients developed RCIE. Among the 29 RCIE patients, 3 were TIA and 26 were ischemic stroke.

### 3.1. Univariate logistic regression analysis for risks of recurrent ischemic events in patients

In the univariate analysis of RCIE in this study, we found that only hypertension was statistically significant in the demographic profile and most factors were statistically significant in the imaging profile, except for CBF <30%. Compared with no patients with no RCIE, ICAS-S/O patients with RCIE had a higher percentage of hypertension (86.2% vs. 62.9%, *p* < 0.027); higher cerebral ischemic volumes, such as *T*_max_ > 6 s volumes (median [IQR]: 140.9 [36.9–223.1] vs. 19.4 [2.5–82.1]; *p* = 0.004), T_max_ > 10 s volumes (median [IQR]: 3.8 [0–25] vs. 0 [0–0.2]; *p* = 0.029); more pronounced mismatch volumes (median [IQR]: 124.4 [36.9–156.4] vs. 10.8 [1.5–73.4]; *p* = 0.009); worse multi-venous scores (median [IQR]: 7 [6–9] vs. 11 [10–12]; *p* < 0.001); lower HIR (median [IQR]: 0.04 [0–0.16] vs. 0 [0–0.03]; *p* = 0.001); and poorer arterial collateral status (median [IQR]: 3 [2–3] vs. 4 [3–4]; *p* = 0.013) (Table 1).

**Table 1:** Univariate logistic regression analysis of patient with recurrent cerebral ischemic events (RCIE).

	No RCIE (*n* = 70)	RCIE (*n* = 29)	Odds ratio	95% CI	*p* value
*Clinical characteristics*					
Age, year, mean ± SD	61.73 (12.77)	62.28 (12.79)	1.003	0.970–1.093	0.845
Male, *n* (%)	47 (67.1)	21 (72.4)	0.778	0.300–2.023	0.607
Smoking, *n* (%)	10 (14.3)	9 (31)	2.700	0.961–7.586	0.059
Hypertension, *n* (%)	44 (62.9)	25 (86.2)	3.693	1.156–11.799	0.027
HDL cholesterol, median (IQR)	1.0 (0.8–1.1)	0.9 (0.7–1.1)	0.254	0.028–2.303	0.223
LDL cholesterol, median (IQR)	2.2 (1.8–2.8)	2.5 (1.8–3.3)	1.322	0.807–2.168	0.268
HbA1c, median (IQR)	5.7 (5.3–6.7)	6.1 (5.7–7.5)	1.263	0.957–1.667	0.098
Admission NIHSS, median (IQR)	1 (0–4)	4 (0–9)	1.061	0.985–1.143	0.118
Discharge NIHSS, median (IQR)	0 (0–3)	2 (0–4)	1.009	0.946–1.076	0.784
*Imaging*					
*T*_max_ > 6 s, median (IQR)	19.4 (2.5–82.1)	140.9 (36.9–223.1)	1.007	1.002–1.012	0.004
*T*_max_ > 10s, median (IQR)	0 (0–0.2)	3.8 (0–25)	1.013	1.001–1.024	0.029
CBF < 30%, median (IQR)	0 (0–4.3)	2 (0–15.1)	1.011	0.997–1.026	0.136
Mismatch, median (IQR)	10.8 (1.5–73.4)	124.4 (36.9–156.4)	1.007	1.002–1.013	0.009
MVS, median (IQR)	11 (10–12)	7 (6–9)	0.022	0.006–0.084	<0.001
HIR, median (IQR)	0 (0–0.03)	0.04 (0–0.16)	0.211	0.083–0.531	0.001
dCTA collaterals, median (IQR)	4 (3–4)	3 (2–3)	0.293	0.112–0.770	0.013
*Location of culprit vessel*, *n* (%)			1.031	0.460–2.313	0.940
ICA	28 (40)	13 (44.8)			
M1	40 (57.1)	16 (55.2)			
ICA + M1	2 (2.9)	0 (0)			
*Cerebral ischemic event*, *n* (%)			1.038	0.190–5.685	0.965
TIA	5 (7.1)	2 (6.9)			
Ischemic stroke	65 (92.9)	27 (93.1)			

IQR, indicates interquartile range; NIHSS, National Institutes of Health Stroke Scale; CBF, cerebral blood flow; *T*_max_, time-to-maximum of the tissue residue function; blood flow; TIA, Transient ischemic attack; SBP, Systolic blood pressure; DBP, Diastolic blood pressure; and HIR, hypoperfusion intensity ratio; CTA, computed tomography angiography; MVS, multi-phase venous score; ICA, internal carotid artery.

### 3.2. Baseline demographics and imaging details dichotomized by mVO profile

Based on ROC analysis, which indicated the best diagnostic performance for mVO in forecasting RCIE (area under the curve, 0.920; 89.7% sensitivity and 84.3% specificity) with an ideal cutoff of MVS 9.5, patients were subcategorized into favorable mVO (mVO+) and unfavorable mVO (mVO−) (Figure 3 in the Data Supplement). The mVO+ group had 62 patients, while the mVO− group included 37 patients. We found that the elements with statistically significant differences in the statistical analysis of the mVO− and mVO+ groups were essentially similar to those in Table 1: hypertension (*p* = 0.018), admission NIHSS (*p* = 0.048), *T*_max_ > 6 s volumes (*p* = 0.042), mismatch volumes (*p* = 0.048), HIR (*p* = 0.007), and dCTA collaterals (*p* < 0.001). Regarding the patient demographics and the results of the medical imaging, no other significant differences were found. The findings are presented in Table 2.

**Table 2:** Baseline demographics and imaging details with mVO.

	mVO+(*n* = 62)	mVO−(*n* = 37)	*p* value
*Clinical demographics*			
Age, mean ± SD	62 (13.7)	52 (11)	0.898
Male, *n* (%)	44 (71)	24 (64.9)	0.526
Smoking, *n* (%)	10 (14.3)	9 (31)	0.316
HDL cholesterol, median (IQR)	1.0 (0.8–1.1)	0.9 (0.7–1.1)	0.306
LDL cholesterol, median (IQR)	2.2 (1.8–2.8)	2.5 (1.8–3.3)	0.355
HbA1c, median (IQR)	5.7 (5.35–6.50)	7.05 (5.85–9.05)	0.458
Hypertension, *n* (%)	38 (61.3)	31 (83.8)	0.018
Hyperlipidemia, *n* (%)	48 (60.8)	14 (70)	0.722
Diabetes, *n* (%)	24 (36.4)	12 (60)	0.505
Admission NIHSS, median (IQR)	1 (0–4)	4 (0–9)	0.048
Discharge NIHSS, median (IQR)	0 (0–3)	2 (0–4)	0.358
Time from admission to dynamic CTP, days, median (IQR)	3 (2–5.5)	4 (2–7)	0.967
*Imaging details*			
*T*_max_ > 6 s, volume (ml), median (IQR)	20.9 (5–86.4)	73.4 (10.1–177.9)	0.042
CBF < 30%, volume (ml), median (IQR)	0(0–4.7)	2 (0–15.1)	0.123
Mismatch, volume (ml), median (IQR)	15.7 (2.9–81)	65.3 (9.2–142.2)	0.048
HIR, median (IQR)	0 (0–0.03)	0.04 (0–0.17)	0.007
dCTA Collaterals, median (IQR)	4 (3–4)	3 (2–3)	<0.001
*Location of culprit vessel*, *n* (%)			0.816
ICA	27 (43.5)	14 (37.8)	
M1	34 (54.8)	22 (59.5)	
ICA + M1	1 (1.6)	1 (2.7)	

mVO, multi-phase venous outflow; IQR, indicates interquartile range; NIHSS, National Institutes of Health Stroke Scale; CBF, cerebral blood flow; *T*_max_, time-to-maximum of the tissue residue function; HIR, hypoperfusion intensity ratio and dCTA, dynamic computed tomography angiography. Scale; CBF, cerebral blood flow; *T*_max_, time-to-maximum of the tissue residue function; blood flow; SBP, Systolic blood pressure; DBP, Diastolic blood pressure; and HIR, hypoperfusion intensity ratio; CTA, computed tomography angiography; MVS, multi-phase venous score; ICA, internal carotid artery.

### 3.3. Multivariate logistic regression analysis for predicting primary outcome (RCIE)

For the main outcome analysis, 99 patients were included into the multivariate logistic regression model. Multivariable ordinal logistic regression indicated that only unfavorable mVO (OR: 2.452; 95% CI: 1.687–3.565; *p* < 0.001) predicted RCIE after adjusting for age, male sex, unfavorable TLC, unfavorable dCTA collaterals, and unfavorable mVO (Table 3).

**Table 3:** Multivariate logistic regression analysis for predicting primary outcome (RCIE).

Predictors	RCIE		CI
	OR	95% CI	*p* value
mVO-(MVS 0–9)	2.592	1.687–4.004	<0.001
Unfavorable TLC	3.082	0.848–11.199	0.087
Unfavorable dCTA collaterals	0.965	0.227–4.110	0.962
Age	1.024	0.931–1.037	0.523
Male	3.180	0.683–14.769	0.140

RCIE, recurrent cerebral ischemic events; TLC, tissue-level collateral; dCTA, dynamic computed tomography angiography; mVO, multi-phase venous outflow and MVS, multi-phase venous score.

### 3.4. Regression analysis for secondary outcome (unfavorable TLC)

When analyzing the secondary results, controlling for unfavorable dCTA collaterals, hypertension, and age, only mVO− (OR: 1.285, 95% CI: 1.090–1.516; *p* = 0.003) predicted unfavorable TLC (Table 4).

**Table 4:** Regression analysis for secondary outcome (unfavorable TLC).

Predictors	Unfavorable TLC		
	OR	95% CI	*p* value
mVO-(MVS 0–9)	2.876	1.075–7.696	0.035
Unfavorable dCTA collaterals	1.86	0.635–5.448	0.258
Hypertension	1.382	0.500–3.824	0.533
Age	0.985	0.951–1.019	0.383

TLC, tissue-level collateral; mVO, multi-phase venous outflow; MVS, multi-phase venous score and dCTA, dynamic computed tomography angiography.

## 4. Discussion

For patients with sICAS-S/O, the risk of cerebral stroke recurrence remains high even after the guidelines for standard secondary stroke prevention therapy are followed. Cerebral hypoperfusion is considered an independent predictor of stroke recurrence in sICAS patients, and perfusion assessment can potentially help screen patients at high risk of stroke recurrence and may provide evidence for further endovascular therapy (8). In this investigation, we examined the correlation between intracranial venous outflow and cerebral tissue perfusion and its impact on the prediction of ischemic stroke recurrence in sICAS-S/O patients. Our results show that an unfavorable intracranial venous outflow was associated with an unfavorable TLC profile (HIR ≤0.01) and predicted a higher risk of RCIE, suggesting that the intracranial venous outflow profile, as an imaging indicator reflecting tissue perfusion, has potential application in predicting the risk of cerebral stroke recurrence in sICAS-S/O patients.

sICAS, which is one of the leading causes of stroke, is characterized by a high stroke recurrence rate and has a relatively insufficient prevention strategy. Most recurrence events took place within 1 month, according to the Mechanisms of Early Recurrence in Intracranial Atherosclerotic Disease, Comparison of Warfarin and Aspirin for Symptomatic Intracranial Arterial Stenosis, and Stenting and Aggressive Medical Management for Preventing Recurrent stroke in Intracranial Stenosis (SAMMPRIS) studies. The second of these studies indicated a recurrence rate as high as 20% even after up to 2 years of observation; our study also demonstrated a high recurrence rate at 1 year in patients with ICAS-S/O (4, 5, 27). Despite secondary prevention measures, patients still had a high recurrence rate following standardized aspirin monotherapy after the initial 90 days of combined antiplatelet treatment. SAMMPRIS showed that approximately 12.2% of patients in the active drug treatment group experienced a recurrent stroke at the end of a 32.4-month (average) follow-up period (5). In addition to drugs, intracranial stenting is one of the optional means of stroke prevention in patients with sICAS who have severe stenosis of large vessels; however, its safety remains a concern. On the one hand, the SAMMPRIS study was forced to stop enrolling patients because of the increased risk of perioperative stroke and death in the PTAS group compared to that in the drug group (5). On the other hand, the 10-year China Angioplasty and Stenting for Symptomatic Intracranial Severe Stenosis study showed that in patients presenting with symptoms of severe cerebral artery stenosis, stenting combined with drug therapy is no less effective than drug therapy alone in averting stroke or mortality, and the study recommended that endovascular therapy should be administered for 3 weeks following the onset of symptoms (28). The high probability of recurrence in sICAS patients is a concern, while preventive treatment measures remain controversial. We hope to discover more effective imaging biomarkers that could improve identification of patients at high risk of cerebral stroke recurrence and possibly aid treatment decisions.

In the past, reperfusion therapy and secondary prevention of ischemic stroke mainly focused on the upstream arteries, while the downstream drainage veins were overlooked. In the cerebral cavity, the intracranial vein accounts for 70–80% of the vascular volume, and it may play an important role in the preservation of brain blood flow and mental function after ischemia (29). It has been proposed that reduced intracranial venous outflow visualization in the cerebral hemisphere affected by cerebral infarction represents a delay in blood flow to the cerebral microcirculation, indicating extensive damage to local brain tissue (30). If venous drainage is severely impaired, arterial blood flowing forward through the brain tissue may be impeded or even blocked from reaching the veins, resulting in blood stasis, while the upstream brain tissue is likely to develop a state of hypoperfusion (20, 21). Intracranial venous outflow may be a better indicator of vascular occlusion tolerance, compare to arterial collateral status, because arterial collateral does not adequately reflect the state of cerebral microcirculation and does not provide a comprehensive assessment of tissue perfusion as it only evaluates the blood flowing into brain tissue, and because venous outflow in this study is a composite score derived from there time observation of the veins, which takes into account the time factor (10, 31). Tobias et al. found that patients with unfavorable venous outflow exhibited poorer collateral circulation and worse cerebral perfusion on baseline imaging than did patients with favorable venous outflow (10, 20). However, it should be noted that not all patients with favorable collaterals had robust venous outflow. Jansen et al. (10) discovered an unfavorable cortical vein opacification score in several patients with favorable artery circulation status, suggesting that impaired autoregulation or other mechanisms may also be involved in venous outflow as well as upstream arterial perfusion (10). Our results showed that patients with an unfavorable mVO (MVS, 0–9) had higher ischemic core volumes, less favorable TLC profiles, and more severe initial symptoms (higher admission NIHSS score). These results imply the importance of intracranial veins in maintaining cerebral perfusion and brain function after ischemia and that intracranial venous outflow may be a potential indicator of cerebral tissue perfusion status.

The predictive role of intracranial venous outflow on acute ischemic stroke prognosis has been well studied, but the predictive value for cerebral stroke recurrence in sICAS-S/O patients remains limited. Cerebral hypoperfusion is considered a valuable predictor of cerebral stroke recurrence in sICAS patients, and venous outflow, as a potential indicator reflecting tissue perfusion status, may also potentially have predictive value for stroke recurrence. Upstream artery severe stenosis or occlusion usually leads to insufficient inflow, and the decrease of upstream artery inflow will inevitably leads to the decrease of downstream outflow, which is manifested as unfavorable venous drainage. Therefore, unfavorable intracranial venous outflow often infers that the patients are in a hypoperfusion state and have a higher risk of recurrent stroke. In this study, we found that most patients with sICAS-S/O who experienced recurrent ischemic stroke presented with multiple infarcts located in the watershed region, which further supports the view that hypoperfusion is related to cerebral stroke recurrence in patients with sICAS. In addition, unfavorable venous outflow was significantly associated with unfavorable tissue perfusion and a higher risk of RCIE. Conversely, no clear independent correlation was found between arterial collateral score and RCIE. Brain tissue perfusion is affected not only by upstream collateral circulation but also by microcirculation and downstream draining veins; therefore, a simple evaluation of arterial collateral circulation cannot accurately reflect tissue perfusion. Intracranial venous outflow is a comprehensive result of the upstream artery, microcirculation, and downstream draining vein, which can better reflect the perfusion status of the cerebral tissue and may be more valuable in predicting the risk of stroke recurrence.

## 5. Limitations

Our study has several limitations. First, this study may have limited generalizability because it was a retrospective observational investigation carried out on a specific group of patients with symptomatic cerebral stenosis in the anterior circulation. Although we strictly followed the guidelines for standard secondary stroke prevention therapy, we still could not rule out the interference of confounding factors, such as differences in the control of vascular risk factors, on stroke recurrence in patients with different venous drainage status. Second, the size of the sample and frequency of RCIE were comparatively small, which may be indicative of bias. In this study, the effect of selection bias was reduced by the following aspects: strict development and implementation of selection and exclusion criteria, collection of perfect data on study subjects as much as possible, and reduction of invalid response rate and lost visit rate. Third, venous variations are present in healthy individuals to a certain degree. Since each patient has physiological differences, our study assessed whether cortical venous outflow had any effect on the recurrence of stroke by examining the symmetry of venous outflow in the affected hemisphere.

## 6. Conclusion

In patients with sICAS-S/O, unfavorable mVO was strongly associated with RCIE and unfavorable TLC. These discoveries support the hypothesis that unfavorable cortical mVO profiles, as measured using MVS, may be valuable imaging biomarkers for predicting RCIE within 12 months.

## Data availability statement

The raw data supporting the conclusions of this article will be made available by the authors, without undue reservation.

## Ethics statement

Written informed consent was obtained from the individual(s) for the publication of any potentially identifiable images or data included in this article.

## Author contributions

JG: conceptualization (lead), data curation (lead), formal analysis (lead), investigation (lead), methodology (equal), validation (equal), writing–original draft (lead), and writing–review and editing (equal). LZ: conceptualization (equal), data curation (equal), formal analysis (equal), writing–original draft (equal), and writing–review and editing (equal). JL: conceptualization (equal) and formal analysis (equal). ZC and XC: data curation (equal) and resources (equal). LH: conceptualization (lead), formal analysis (equal), investigation (equal), methodology (equal), project administration (equal), resources (equal), supervision (lead), writing–original draft (lead), and writing–review and editing (lead). MY and JY: data curation (equal) and investigation (equal). All authors contributed to the article and approved the submitted version.

## Funding

This work was supported by Science and Technology Projects in Guangzhou (Grant Number 202201020062).

## Conflict of interest

The authors declare that the research was conducted in the absence of any commercial or financial relationships that could be construed as a potential conflict of interest.

## Publisher’s note

All claims expressed in this article are solely those of the authors and do not necessarily represent those of their affiliated organizations, or those of the publisher, the editors and the reviewers. Any product that may be evaluated in this article, or claim that may be made by its manufacturer, is not guaranteed or endorsed by the publisher.
